# DNA methylation profiling of ovarian carcinomas and their *in vitro *models identifies *HOXA9*, *HOXB5*, *SCGB3A1*, and *CRABP1 *as novel targets

**DOI:** 10.1186/1476-4598-6-45

**Published:** 2007-07-10

**Authors:** Qinghua Wu, Ragnhild A Lothe, Terje Ahlquist, Ilvars Silins, Claes G Tropé, Francesca Micci, Jahn M Nesland, Zhenhe Suo, Guro E Lind

**Affiliations:** 1 Department of Pathology, Rikshospitalet-Radiumhospitalet Medical Center, Oslo, Norway; 2 Department of Cancer Prevention, Institute for Cancer Research, Rikshospitalet-Radiumhospitalet Medical Center, Oslo, Norway; 3 Centre for Cancer Biomedicine, University of Oslo, Oslo, Norway; 4 Department of Gynecologic Oncology, Rikshospitalet-Radiumhospitalet Medical Center, Oslo, Norway; 5 Department of Medical Genetics, Rikshospitalet-Radiumhospitalet Medical Center, Oslo, Norway

## Abstract

**Background:**

The epigenetics of ovarian carcinogenesis remains poorly described. We have in the present study investigated the promoter methylation status of 13 genes in primary ovarian carcinomas (n = 52) and their *in vitro *models (n = 4; ES-2, OV-90, OVCAR-3, and SKOV-3) by methylation-specific polymerase chain reaction (MSP). Direct bisulphite sequencing analysis was used to confirm the methylation status of individual genes. The MSP results were compared with clinico- pathological features.

**Results:**

Eight out of the 13 genes were hypermethylated among the ovarian carcinomas, and altogether 40 of 52 tumours were methylated in one or more genes. Promoter hypermethylation of *HOXA9*, *RASSF1A*, *APC*, *CDH13*, *HOXB5*, *SCGB3A1 (HIN-1)*, *CRABP1*, and *MLH1 *was found in 51% (26/51), 49% (23/47), 24% (12/51), 20% (10/51), 12% (6/52), 10% (5/52), 4% (2/48), and 2% (1/51) of the carcinomas, respectively, whereas *ADAMTS1*, *MGMT*, *NR3C1*, *p14*^*ARF*^, and *p16*^*INK*4*a *^were unmethylated in all samples. The methylation frequencies of *HOXA9 *and *SCGB3A1 *were higher among relatively early-stage carcinomas (FIGO I-II) than among carcinomas of later stages (FIGO III-IV; *P *= 0.002, *P *= 0.020, respectively). The majority of the early-stage carcinomas were of the endometrioid histotype. Additionally, *HOXA9 *hypermethylation was more common in tumours from patients older than 60 years of age (15/21) than among those of younger age (11/30; *P *= 0.023). Finally, there was a significant difference in *HOXA9 *methylation frequency among the histological types (*P *= 0.007).

**Conclusion:**

DNA hypermethylation of tumour suppressor genes seems to play an important role in ovarian carcinogenesis and *HOXA9*, *HOXB5*, *SCGB3A1*, and *CRABP1 *are identified as novel hypermethylated target genes in this tumour type.

## Background

Ovarian cancer is often not detected until it has reached an advanced stage and is therefore among the most lethal gynaecological cancer diseases. Tumour stage at diagnosis, residual disease following cytoreductive surgery, and performance status which is evaluated by Karnofsky Index [1] are the three major prognostic factors [2]. Epithelial ovarian carcinoma accounts for over 90% of all cases and includes the following major histological subtypes: serous-, mucinous-, endometrioid-, and clear cell- carcinomas. In Norway, more than 90% of patients with ovarian carcinoma are older than 40 years, with a peak incidence at the age of 75–79 [3].

A number of genetic changes have been shown to accumulate during carcinogenesis, including DNA copy number changes and various types of gene mutations. Simultaneously, several epigenetic changes have been shown to be present and possibly participate in the carcinogenesis [4,5]. DNA methylation is a well-studied epigenetic mechanism, defined as a heritable and enzyme-induced chemical modification of DNA, not altering the DNA sequence [6]. In higher order eukaryotes, cytosines located 5' to guanosines in so-called CpG sites are the targets for methylation and a high density of such sites within a limited stretch of DNA constitutes a CpG island [7]. Approximately half of the human genes contain such CpG islands in their 5' regulatory sequence, and DNA hypermethylation of this region is associated with lost or reduced gene expression, representing an important alternative mechanism for the inactivation of tumour suppressor- and DNA repair- genes. Among ovarian carcinomas, only a handful such genes have been shown to exhibit DNA promoter hypermethylation, including the Insulin-like growth factor binding protein-3 (*IGFBP-3*) [8], the Deleted in Lung and Esophageal Cancer 1 (*DLEC1*) [9], and the breast cancer gene *BRCA1 *[10].

Some genes, like the cell cycle inhibitor *p16*^*INK*4*a *^(*CDKN2A*) and estrogen receptor (*ER*), are frequently hypermethylated across several cancer types, whereas others are more common in specific cancer types, such as hypermethylated *DAPK *in lung cancer and lymphoma, and *GSTP1 *in prostate-, breast-, kidney-, and liver cancer [11]. Hence, hypermethylation of target genes seems to be tumour-type specific and can potentially be used in the clinic to detect neoplasms, predict tumour response, and develop therapies that target hypermethylated tumour suppressor genes [12,13]. Here, we present the DNA methylation profile of 13 genes in a series of ovarian carcinomas and cancer cell lines. The investigated gene promoters were chosen from loci previously reported to be methylated in ovarian cancer (n = 7; *APC*, *CDH13*, *MGMT*, *MLH1*, *p14*^*ARF*^, *p16*^*INK*4*a*^, *RASSF1A*) and from loci methylated in other tumour types (n = 6; *ADAMTS1*, *CRABP1*, *HOXA9*, *HOXB5*, *NR3C1*, *SCGB3A1*).

## Results

### Gene promoter methylation in ovarian carcinomas

**Figure 1:**
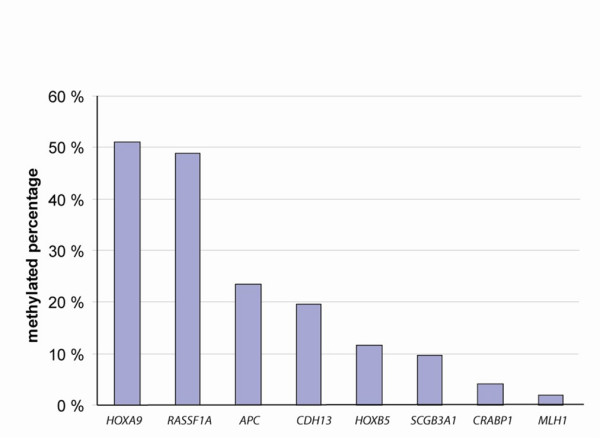
**Methylation profile of primary ovarian carcinomas**. Thirteen genes were analyzed by methylation-specific polymerase chain reaction (MSP) in 52 ovarian carcinomas. *ADAMTS1*, *MGMT*, *NR3C1, p14*^*ARF*^, and *p16*^*INK*4*a *^were unmethylated in all tumours samples analyzed and are excluded from the figure. Axis Y represents the promoter methylation percentage of individual genes.

**Figure 2:**
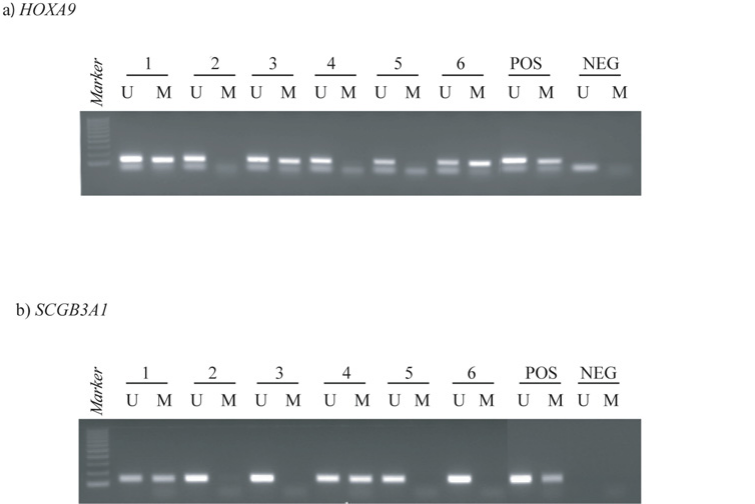
**Representative methylation-specific polymerase chain reaction (MSP) results from primary ovarian carcinomas**. PCR products in lane U indicate the presence of unmethylated alleles whereas PCR products in lanes M indicate the presence of methylated alleles. Panel A illustrates *HOXA9 *(the upper bands represent MSP products, whereas the lower bands are excess primers). Panel B illustrates *SCGB3A1*. Abbreviation: Pos, positive control (DNA from normal blood is used as control for unmethylated samples, and *in vitro *methylated DNA is used as control for methylated samples); Neg, negative control (template replaced by water); Lane 1–6, individual ovarian carcinomas; 100 bp DNA marker from Promega Corp, Madison, WI, USA. The illustration has been processed from a photo including more samples. Hence, the controls have been moved from their original position and pasted adjacent to the selected samples shown here.

Eight out of the 13 genes analyzed by methylation-specific polymerase chain reaction (MSP) showed promoter hypermethylation in one or more of the primary ovarian carcinomas (n = 52), and altogether 77% (40/52) of these tumours harboured promoter hypermethylation in at least one of these eight genes. The results are summarized in Table 1 and Figure 1, and representative MSP gel bands are presented in Figure 2. *HOXA9 *and *RASSF1A *were hypermethylated in high frequencies (26/51 51% and 23/47 49%, respectively), *APC *and *CDH13 *at intermediate frequencies (12/51 24% and 10/51 20%, respectively), whereas *HOXB5*, *SCGB3A1*, *CRABP1 *and *MLH1 *were less frequently methylated (6/52 12%, 5/52 10%, 2/48 4%, and 1/51 2%, respectively). No methylation was detected in *ADAMTS1*, *MGMT*, *NR3C1*, *p14*^*ARF*^, or *p16*^*INK*4*a*^. Furthermore, benign (n = 2) and borderline (n = 2) ovarian tumours were unmethylated for all analyzed genes.

**Table 1:** Methylation correlated with clinical features in ovarian carcinomas

	***APC***	***CDH13***	***CRABP1***	***HOXA9***	***HOXB5***	***MLH1***	***RASSF1A***	***SCGB3A1***
**Age group**								
<60 years	6/29	6/30	0/29	11/30	2/30	1/30	15/26	1/30
> = 60 years	6/22	4/21	2/19	15/21	4/22	0/21	8/21	4/22
*P *value	NS	NS	NS	0.023	NS	NS	NS	NS
**FIGO stage**								
IA-IIC	9/25	7/24	2/24	18/24	2/25	1/24	14/23	5/25
III-IV	3/26	3/27	0/24	8/27	4/27	0/27	9/24	0/27
*P *value	0.052	NS	NS	0.002	NS	NS	NS	0.020
**Histological Type**								
Serous	3/19	1/19	0/19	4/19	2/19	0/19	8/19	0/19
Mucous	3/5	1/4	0/4	3/4	0/5	0/4	2/5	3/5
Clear cell	3/5	3/5	2/3	4/5	1/5	0/5	4/5	2/5
Endometrioid	3/17	3/17	0/16	12/17	2/17	1/17	7/14	0/17
*P *value	NS	0.041	<0.001	0.007	NS	NS	NS	<0.001
**Grade of differentiation**								
Poorly	5/23	4/23	2/21	11/23	3/23	0/23	8/22	2/23
Moderate	3/14	4/15	0/14	9/15	3/15	1/15	10/13	0/15
Well	4/12	1/11	0/11	5/11	0/12	0/11	4/10	3/12
*P *value	NS	NS	NS	NS	NS	NS	0.055	0.095

Abbreviations: NS, no significance; *P *values are two-sided and considered statistically significant when *P *<= 0.05. The table is based on 52 primary carcinomas from 50 patients.

### Gene promoter methylation in ovarian cancer cell lines

The detailed methylation status for each cell line is shown in Table 2. *APC*, *CDH13*, *CRABP1*, *HOXA9*, *HOXB5*, *RASSF1A*, and *SCGB3A1 *were found to be hypermethylated both in the primary tumours and in ovarian cancer cell lines, whereas *ADAMTS1 *and *MGMT *were hypermethylated only among cell lines. Finally, *MLH1*, *NR3C1*, *p14*^*ARF*^, and *p16*^*INK*4*a *^were unmethylated in all cell lines.

**Table 2:** Methylation status in ovarian cancer cell lines

	ES-2	OV-90	OVCAR-3	SKOV-3
*ADAMTS1*	U	U/M	U	U
*APC*	U	U/M	U	U
*CDH13*	U	U	U	U/M
*CRABP1*	M	U	U	U/M
*HOXA9*	M	M	U/M	M
*HOXB5*	U/M	U/M	U	U
*MGMT*	U/M	U	U	U
*MLH1*	U	U	U	U
*NR3C1*	U	U	U	U
*p14*^*ARF*^	U	U	U	U
*p16*^*INK*4*a*^	U	U	U	U
*RASSF1A*	U	M	M	M
*SCGB3A1*	U	U	U/M	U/M

Abbreviation: U, unmethylated; M: methylated.

### Verification of DNA promoter methylation by bisulphite sequencing

**Figure 3:**
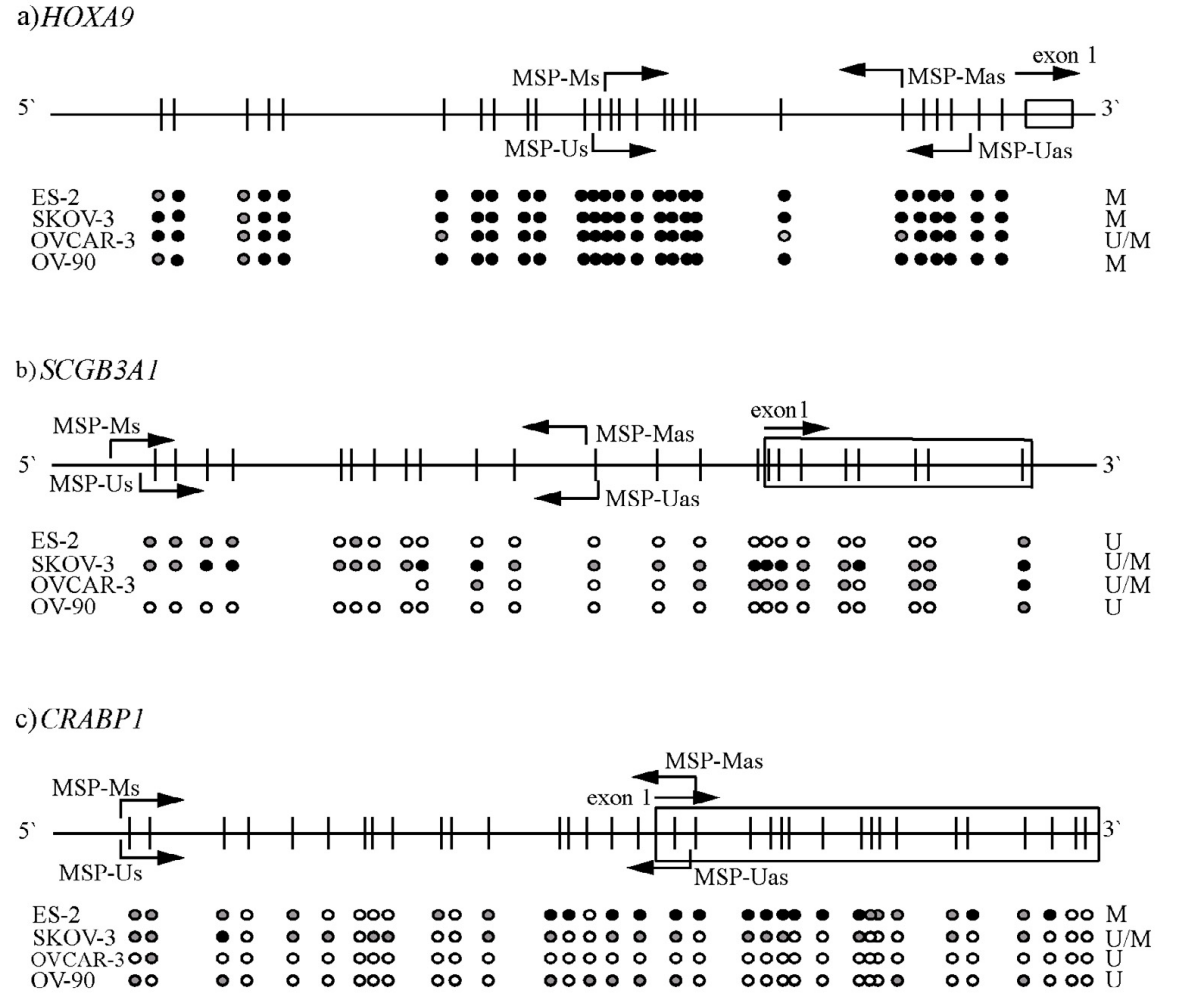
**Direct bisulphite sequencing verified methylation status as assessed by methylation-specific polymerase chain reaction (MSP)**. Methylation status of individual CpG sites in *HOXA9 (a), SCGB3A1 (b)*, and *CRABP1 (c)*. The upper panel of each gene shows the CpG sites (vertical bars) amplified by the bisulphite sequencing primers. Bent arrows indicate the location of the MSP primers, whereas straight arrows indicate the transcription start site of individual genes. Black filled circles represent methylated CpGs; Open circles represent unmethylated CpGs; Gray circles represent partial methylation, defined as 21–80% methylation. The column at the right side of each panel (U, M, and U/M) shows the methylation status as assessed by MSP analysis.

**Figure 4:**
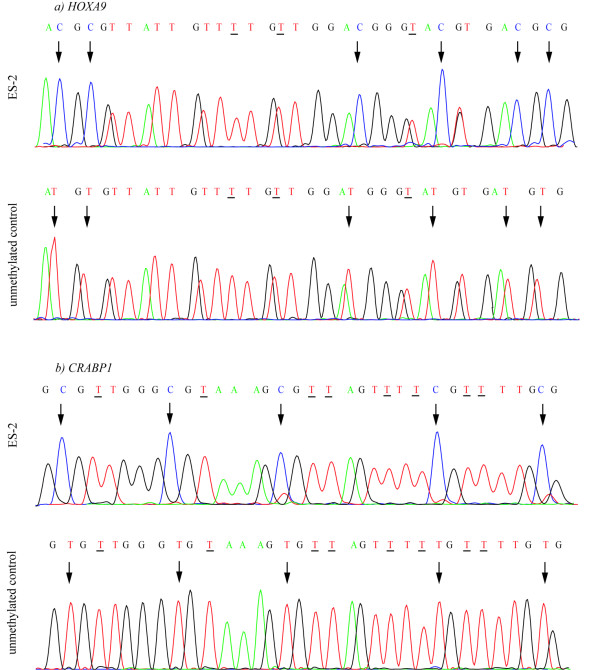
**Bisulphite sequence electropherograms**. Panel a) illustrates CpG sites 9–14 from the *HOXA9 *bisulphite sequencing fragment in the methylated ES-2 cell line (top) and unmethylated control (bottom). Panel b) illustrates CpG sites 15–19 from the *CRABP1 *bisulphite sequencing fragment also from the methylated ES-2 cell line and unmethylated control. CpG sites are indicated by arrows, whereas originally Cs converted to Ts by bisulphite sequencing are underlined. For both genes, bisulphite treated normal blood was used as unmethylated control.

*CDH13*, *CRABP1*, *HOXA9*, and *SCGB3A1 *were subjected to direct bisulphite sequencing in the four ovarian carcinoma cell lines. In general, there was a good concordance between MSP status and bisulphite sequences, limiting the potential detection of false positives by the first method. The results are summarized in Figure 3 and representative electropherograms are displayed in Figure 4.

### Methylation profile compared with clinical characteristics

Patients over and under 60 years of age (mean across all patients = 58 years) displayed a similar distribution regarding FIGO stage, histology, and promoter methylation frequencies, with the exception of hypermethylated *HOXA9*, which was significantly more frequent in tumours from older patients (*P *= 0.023; Table 1).

By comparing the distribution of histological type and clinical stage, we found that 14 out of 17 carcinomas of the endometrioid type were of FIGO stages I to II and 18 out of 19 carcinomas of the serous histotype were of FIGO stages III to IV. With the exception of *HOXB5*, higher promoter methylation frequencies were found among tumours from patients with relatively early FIGO stages (I-II) than late stages, but reached statistical significance only for *HOXA9 *and *SCGB3A1 *(*P *= 0.002, *P *= 0.020, respectively). The same trend could also be seen for *APC *(*P *= 0.052).

Across all genes analyzed, the serous subtype displayed the lowest frequencies of hypermethylation, whereas the clear cell tumours displayed the highest (*P *= 0.078, Kruskal-Wallis test). At the single gene level, this was significant for *CDH13*, *CRABP1*, and *HOXA9 *(*P *= 0.041, *P *< 0.001, *P *= 0.007, respectively). Further, the promoter of the *SCGB3A1 *gene was only hypermethylated among mucous and clear cell tumours (*P *< 0.001).

No association could be seen between methylation frequency and grade of tumour differentiation.

Finally, four ovarian carcinomas showed simultaneous methylation of four or more out of the 13 genes analyzed. Three of these tumours were of the clear cell histotype, whereas the last tumour was endometrioid. The remaining carcinomas displayed in average 1.4 methylated genes.

### Methylation profiles compared with microsatellite instability (MSI)

In this study, microsatellite instability (MSI) status was analyzed in 54 samples including 50 carcinomas, 2 borderline, and 2 benign tumours. MSI was seen in a single carcinoma of mixed histotype. Further, seven ovarian carcinomas were MSI-low, significantly associated with the clear cell and endometrioid histotype (*P *= 0.013). The remaining samples (n = 46) were microsatellite stable (MSS). MSI status was not associated with any additional clinical parameters, such as FIGO stage, differentiation grade or age, although MSI-low carcinomas had a higher methylation frequency of *HOXB5 *than did the MSS tumours (*P *= 0.006).

Among the four ovarian cancer cell lines, SKOV-3 was MSI whereas the other cell lines displayed a MSS phenotype.

## Discussion

Our data support the view that promoter hypermethylation is a common mechanism involved in ovarian carcinogenesis and four target genes, *HOXA9*, *HOXB5*, *SCGB3A1*, and *CRABP1*, novel to this cancer type are identified.

Homeodomain-containing (HOX) genes encode transcriptional factors functioning during embryonic development to control patterning, differentiation, and proliferation (see review [14]). In mouse development, *HOXB5 *shows tissue specific methylation in the adult, but is unmethylated in the fetal tissues [15]. High methylation percentage of *HOXA9 *has been reported in early stages of primary squamous cell carcinomas of the lung [16]. In the present study, the promoter of *HOXA9 *was frequently hypermethylated and associated with early-stage (FIGO stage I-II) ovarian carcinomas. Interestingly, the majority of these tumours were of the endometrioid histotype. *HOXA9 *hypermethylation was also present in the other histological subtypes, including the serous type, which across all analyzed genes displayed the lowest frequency of promoter methylation. No normal ovarian samples were included in the present study. The proper normal control would be the epithelial lining of the ovaries, which consists of a single cell layer. However, the lack of methylation in benign and borderline tumours as well as in blood indicates that the *HOXA9 *promoter is unmethylated in the normal situation. Hence, aberrant gene expression of *HOXA9 *may be involved in the molecular pathway of ovarian carcinogenesis. A previous study has suggested that the development of various histotypes of ovarian cancer in mice is partly dependent on the ectopic expression levels of *HOXA9 *[17], however, additional studies are needed before this can be concluded. In the present study, *HOXA9 *promoter methylation was more frequent in tumours from older patients than in tumours from younger patients. Aging is one the most important risk factors for development of neoplasia and methylation has previously been shown to increase with age [18,19]. The peak incidence of ovarian cancer is over 60 years, and aging can therefore not be excluded as a contributor to *HOXA9 *hypermethylation in this disease.

*SCGB3A1*, also named *HIN1 *(high in normal-1), encodes a small secreted protein, secretoglobin 3A1, and belongs to the secretoglobin family [20]. It is reported to be a potent inhibitor of anchorage-dependent and anchorage-independent cell growth, cell migration, and invasion [21]. Hypermethylation-induced down-regulation of this gene has been found in several cancer types, such as breast-, non-small cell lung-, small cell lung-, colorectal-, and testicular- cancer, suggesting a potential tumour suppressor function [22,23]. We demonstrate here that *SCGB3A1 *promoter hypermethylation also occurs in ovarian carcinomas, suggesting that this event plays a role in the development of a subgroup of these tumours.

Recently, we showed that promoter hypermethylation of *ADAMTS1*, *CRABP1*, and *NR3C1 *was frequent among colorectal carcinomas and cell lines and to a certain extent in colorectal adenomas [24]. Since ovarian cancer also belongs to the hereditary non-polyposis colorectal cancer (HNPCC) tumour spectrum and the fact that sporadic tumours of the same type often exhibit the same molecular aberrations, these three genes were included in the present study. *CRABP1*, which is a member of a family of small cytosolic lipid binding proteins and encodes a cellular retinoic acid binding protein [25], was the only gene in which hypermethylation was found and then only in two clear cell tumours. *ADAMTS1*, a metalloproteinase of the ADAM family [26], was found methylated in a single OV-90 cell line but not in ovarian carcinomas. The CpG island in the promoter of *NR3C1 *remained unmethylated in all the samples. These findings, and the fact that neither prostate-, kidney-, nor testicular- cancer showed methylation of these genes [24], support the hypothesis that *ADAMTS1*, *CRABP1*, and *NR3C1 *are targeted by hypermethylation preferentially in colorectal tumours.

Additionally, we analyzed seven genes which have previously been reported to be hypermethylated in ovarian cancer, including *APC*, *CDH13*, *MGMT*, *MLH1*, *p14*^*ARF*^, *p16*^*INK*4*a*^, and *RASSF1A*. For *RASSF1A *[27,28], *APC *[29,30], and *CDH13 *[31], we found comparable methylation frequencies to previous reports, which have also shown that promoter hypermethylation of these genes are associated with loss of gene expression in various tumour types [31-33]. The methylation of *p16*^*INK*4*a *^has been shown to be important in tumour development in several tissue types, such as the colon, lung, head and neck, pancreas, cervix, large bowel, leukemia and lymphoma but is seemingly not important in ovarian cancer [34-36]. Here we find that both *p14*^*ARF *^and *p16*^*INK*4*a *^were unmethylated in all samples analyzed, which is in agreement with most other reports [35,37]. However, McCluskey *et al*. [38] identified a high frequency of *p16*^*INK*4*a *^methylation in ovarian tumours of low malignant potential, compared with malignant carcinomas.

Approximately, seventy percent of the microsatellite unstable sporadic colorectal carcinomas results from loss of MLH1 caused by DNA promoter hypermethylation [39]. In ovarian carcinomas, methylation of *MLH1 *is less common and has been reported only among clear cell and endometrioid subtypes [40]. In the present study, only one ovarian carcinoma, belonging to the endometrioid subtype, harboured promoter hypermethylation of *MLH1*. The methylation did not lead to loss of *MLH1 *expression since no sign of a MSI phenotype was shown in this tumour, indicating that at least one allele is likely to stay unaffected. Similarly, *MGMT*, a second repair enzyme encoding gene, was methylated in a single cell line ES-2, established from a human primary clear cell carcinoma, but not in any clinical samples. Although methylation of *MLH1 *and *MGMT *was uncommon in the present study, we cannot rule out that epigenetic inactivation of DNA repair genes may be a mechanism associated with specific subtypes of ovarian cancer.

In colorectal tumours, a CpG island methylator phenotype (CIMP) has been suggested, characterized by frequent promoter hypermethylation [41] and associated with the MSI phenotype [42]. The concept of CIMP has also been demonstrated in other cancer types, such as gastric cancer [43]. From the present findings, CIMP does not seem to be common among ovarian carcinomas, as only few tumours showed simultaneous hypermethylation of several genes. However, CIMP is not merely defined by the methylation frequency of randomly selected genes, but rather by assessing the methylation status of a specific gene panel [44]. Hence, further studies including more MSI positive samples are required to shed light on CIMP in ovarian carcinomas. The frequently methylated ovarian carcinomas identified here belonged to the clear cell- and endometrioid histotype, suggesting that wide-spread methylation may be associated with distinct subgroups of ovarian tumours.

The five-year survival rate for patients with early stage ovarian cancer (FIGO stage I-II) and advanced stage cancer (FIGO stage III-IV) is 72% and 27%, respectively [45]. Unfortunately, most women receive their diagnosis at a late stage when the chance of cure is low [46], which might in part be explained by the non-specific nature of the symptoms [47]. Identification of biomarkers for early detection of disease could therefore significantly improve the survival rate among these patients. Methylated DNA can be detected in various body fluids from patients with neoplasia and the methylation status of individual as well as panels of genes can potentially be used for risk assessment [48]. DNA methylation has previously been identified in both serum and peritoneal fluid from ovarian cancer patients [49,50]. In the present study, we find high methylation frequencies of *HOXA9 *and *RASSF1A *in tumours from this patient group. If the promoter methylation can be detected also in body fluids from these patients, the genes represent epigenetic markers that might be included in a non-invasive diagnostic test. Even though *APC *and *SCGB3A1 *promoter methylation is less frequent, it is nevertheless associated with early stage ovarian tumours and might also have a diagnostic potential. An association between high promoter methylation frequencies and early stage ovarian carcinomas has recently also been shown by Tam *et al*. [51].

## Conclusion

CpG island promoter hypermethylation of tumour suppressor genes is a common event in primary ovarian carcinomas and cell lines, and *HOXA9, HOXB5*, *SCGB3A1*, and *CRABP1*, represent novel hypermethylated target genes in this disease.

## Methods

### Tissue samples

Fifty-six fresh frozen ovarian tissue samples, surgically removed between 1993 and 2005, were collected from a tissue bank at Department of Pathology, Rikshospitalet-Radiumhospitalet Medical Center, University of Oslo. Fifty-two were carcinomas, including nineteen tumours of the serous histotype, five of mucous, five of clear cell, seventeen of endometrioid, and six of mixed histotype. In addition, two benign and two borderline ovarian tumours were included in the present study (Table 1). The study has been approved by The Regional Committee for Medical Research Ethics South of Norway (S-06277a), The Social and Health Directorate (06/3280), and The Data Inspectorate (06/5345).

### Ovarian carcinoma cell lines

Four ovarian carcinoma cell lines, ES-2, OV-90, OVCAR-3, and SKOV-3 (American Type Culture Collection, Manassas, USA) were included in the present study. The ES-2 cell line has originally been derived from a poorly differentiated ovarian clear cell carcinoma with fibroblast morphology. The remaining cell lines have been cultured from malignant ascites from patients with adenocarcinoma. All cell lines were cultured in RPMI medium with 10% fetal bovine serum, 2 mM L-Glutamine, 100 units/ml penicillin, and 100 ug/ml streptomycin. All reagents are from Cambrex Bio Science Verviers in Belgium.

### DNA extraction

Genomic DNA from fresh frozen ovarian cancer specimens and ovarian carcinoma cell lines was extracted using the 340A Nucleic Acid Extractor (Applied Biosystems, Foster City, CA, USA), applying standard phenol/chloroform extraction followed by ethanol precipitation.

### Bisulphite modification of DNA and methylation-specific polymerase chain reaction (MSP)

DNA from all samples was treated with sodium bisulphite, which converts all unmethylated cytosines to uracils, whereas methylated cytosines remain unchanged [52]. Briefly, 1.3 μg of DNA was denatured by incubation with 0.3 M NaOH for 15 min at 37°C. Hydroquinone (Sigma Chemical Co., St. Louis, MO, USA) and sodium bisulphite (Sigma Chemical Co, USA) at pH 5.0 were added to the samples to a final concentration of one mM and 3.7 M, respectively, prior to incubation at 50°C for 16 h. Bisulphite treated DNA was purified using the Wizard DNA clean-up kit (Promega Corp, Madison, WI, USA) and eluted in 100 μL MQ water. In order to complete the conversion of unmethylated cytosines, NaOH was added to a final concentration of 0.3 M and the samples were incubated for 15 minutes at 37°C. Modified DNA was precipitated with 100% ethanol, 10 μg glycogen, and 0.3 M AcNH4 at minus 80°C overnight, then re-suspended in 30 μl MQ water, and stored at 4°C. Thirteen genes were subjected to MSP [53], more specifically *ADAMTS1*, *APC*, *CDH13*, *CRABP1*, *HOXA9*, *HOXB5*, *MGMT*, *MLH1*, *NR3C1*, *p14*^*ARF*^, *p16*^*INK*4*a*^, *RASSF1A*, and *SCGB3A1*. Since bisulphite modification leads to sequence differences, two pairs of primers were used to amplify each gene (see Additional file 1), one specific for unmethylated template and the other specific for methylated template [53]. The 25 μl PCR mixture contained 1 × PCR buffer, 1.0–1.5 mM MgCl_2_, 20 pmol of each primer, 200 μM dNTP, and 0.625–1.0 U HotStarTaq DNA Polymerase (Qiagen, Valencia, CA). All MSP reactions were run twice, and a third independent MSP round was performed when the results were not concordant. Human placental DNA (Sigma Chemical Co, St. Louis, MO, USA) treated *in vitro *with *SssI *methyltransferase (New England Biolabs Inc., Beverly, MA, USA) was used as a positive control for the methylated MSP reaction, whereas DNA from normal lymphocytes was used as a positive control for unmethylated alleles. Water was used as a negative PCR control in both reactions.

### Bisulphite sequencing

With the use of bisulphite sequencing, original 5-methyl cytosines can be detected as cytosines in the sequence, whereas unmethylated cytosines will be converted to uracils and amplified as thymines [54]. *CDH13, CRABP1, HOXA9*, and *SCGB3A1 *(primers are given in Additional file 1) were subjected to direct bisulphite sequencing in the four ovarian carcinoma cell lines. The fragments were amplified with HotStarTaq DNA Polymerase, and excess primer and nucleotides were removed by ExoSAP-IT treatment following the manufacturer's protocol (GE Healthcare, USB Corporation, Ohio, USA). The purified products were subsequently sequenced using the dGTP BigDye Terminator Cycle Sequencing Ready Reaction kit (Applied Biosystems, Foster City, CA, USA) in an ABI Prism 3730 Sequencer (Applied Biosystems). The approximate ratio of methyl cytosine present in each CpG site was calculated by dividing the peak height of the cytosine signal with the sum of the cytosine and thymine peak height signals, as previously described [55]. CpG sites with ratios from 0–0.2 were classified as unmethylated, ratios from 0.21 – 0.80 were classified as partially methylated, and ratios from 0.81 – 1.0 were classified as methylated.

### Microsatellite instability (MSI)

MSI status was determined in all samples using the consensus panel of five microsatellite markers (BAT25, BAT26, D2S123, D5S346, and D17S250) [56] (see Additional file 1). A tumour was considered to be MSI-high if two or more of the five markers exhibited novel alleles compared to normal DNA, MSI-low if only one marker deviated from the normal pattern, and microsatellite stable (MSS) if none of the tumour genotypes showed an aberrant pattern. Control DNA corresponding to the individual tumours was not available from this patient series and thus single allele changes, *i.e*. the presence of two alleles, can reflect the heterozygote constitutional genotype or a homozygote with a novel tumour specific allele. Thus, dinucleotide markers were not scored when such a pattern appeared in the tumours.

Thirty-seven ng DNA template was amplified in pentaplex, in a ten μl reaction volume consisting of 1 × Multiplex PCR Mastermix (containing buffer, 1.5 mM MgCl_2_, nucleotides, and enzyme; QIAGEN GmbH, Hilden, Germany), 1.2 pmol BAT25 primers (sense primer labeled with NED in the 5' end), 1.6 pmol BAT26 primers (sense primer labeled with 6-FAM in the 5' end), 1.6 pmol D2S123 primers (sense primer labeled with NED in the 5' end), 1.2 pmol D5S346 primers (sense primer labeled with VIC in the 5' end), 3 pmol D17S250 primers (sense primer labeled with 6-FAM in the 5' end; Applied Biosystems, Foster City, CA, USA). The PCR annealing temperature was 55°C and the program included 27 cycles.

From these PCR products, 0.5 μl was mixed with 0.5 μl GeneScan™ 500 LIZ^® ^Size Standard (Applied Biosystems) and 9 μl deionized formamide (Kodak Eastman Chemical Company, New Haven, CT, USA). The samples were subsequently denatured and separated by capillary electrophoresis on a 48-capillary 3730 DNA Analyzer (Applied Biosystems, Foster City, CA, USA). Allelic sizes were determined using GeneMapper 3.7 software (Applied Biosystems) and the results were independently scored by two investigators. A second round of analyses confirmed the results.

### Statistical Analysis

All 2 × 2 contingency tables were analyzed using Fisher's exact test. 3 × 2 and 4 × 2 tables were analyzed using Chi square analysis. The potential association between methylation frequencies across all genes analyzed and tumour histology was analyzed by Kruskal-Wallis test (SPSS, version 11.5). *P *values were derived from two-tailed statistical tests and *P *<= 0.05 were considered to be statistically significant.

## Competing interests

The author(s) declare that they have no competing interests.

## Authors' contributions

All authors have read and approved the final version of the manuscript.

QW carried out the MSP analyses and the bisulphite sequencing, interpreted the results, performed the statistics, and drafted the manuscript. RAL conceived the study, participated in its design, and contributed with scientific discussion and manuscript preparation. TA ran MSI analyses and contributed to manuscript preparation. IS, CGT, and FM provided clinic data and samples. ZHS and JMN contributed to sample preparation, pathological diagnosis, scientific discussion and manuscript preparation. GEL participated in the design of the study and was responsible for its coordination, provided the epigenetic techniques, interpreted the results independently of author 1, reviewed all statistics, and contributed in the preparation of the manuscript.
